# Barriers to recruitment when conducting a commissioned randomised controlled trial of medication versus psychological therapy for generalised anxiety disorder: some lessons learned

**DOI:** 10.1186/s13063-019-3385-5

**Published:** 2019-05-24

**Authors:** Anastasia K. Kalpakidou, John Cape, Tarun J. Limbachya, Irwin Nazareth, Marta Buszewicz

**Affiliations:** 1 Marie Curie Palliative Care Research Department (MCPCRD), University College London, Division of Psychiatry, 6th Floor, Wing A, Maple House, 149 Tottenham Court Road, London, W1T 7NF UK; 2 Research Department of Clinical, Educational & Health Psychology, University College London, Division of Psychology & Language Sciences, 1-19 Torrington Place, London, WC1E 7HB UK; 3 University of East London, Department of Clinical Psychology, Stratford Campus, Water Lane, London, E15 4LZ UK; 4 Research Department of Primary Care & Population Health (PCPH), University College London, Institute of Epidemiology & Health Care, Upper 3rd Floor, Rowland Hill Street, London, NW3 2PF UK

**Keywords:** Randomised controlled trial, Barriers to recruitment, Generalised anxiety disorder, Medication, Psychological therapy

## Abstract

**Background:**

Poor recruitment is the most common reason for premature discontinuation of randomised controlled trials (RCTs). An RCT of medication versus psychological therapy for generalised anxiety disorder (GAD) was discontinued prematurely by the UK National Institute of Health Research funders because of recruitment failure. In order to inform future research studies, this article explores the reasons for poor recruitment and aspects which could have been improved.

**Methods:**

The trial recruited participants via psychological well-being practitioners (PWPs) employed within local Improving Assess to Psychological Therapies (IAPT) services at four sites in England. For this study, we initially examined the recruitment data to identify reasons why potential participants were reluctant to participate in the trial. We then investigated reasons the PWPs did not identify more potential participants. Finally, we performed retrospective analyses of a computerised clinical records system used by the IAPT services in this study. These analyses aimed to establish the number of potential participants who had not been approached about the trial as well as whether there were additional factors affecting the numbers of people who might be eligible to take part. Data were obtained for all patients assessed during the period from the date on which recruitment commenced until the closure of the trial.

**Results:**

Three quarters of those patients identified as possibly suitable for the trial declined to take part; the great majority did so because they did not want to be randomly assigned to receive medication. Our retrospective database analyses showed that only around 12% of potentially eligible patients for the trial were identified by the PWPs at the pilot sites. The results also indicated that only 5% of those noted at entry to the IAPT services to have a score of at least 10 on the GAD-7 questionnaire (a self-completed questionnaire with high sensitivity and specificity for GAD) would have been eligible for the trial.

**Conclusions:**

Our findings suggest that poor recruitment to RCTs can be significantly affected by participants’ treatment preferences and by factors influencing the recruiting clinicians. It may also be important not to include too many restrictions on inclusion criteria for pragmatic trials aiming for generalisable results.

**Trial registration:**

ISCRTN14845583. Registration date: 5 February 2015.

## Background

Randomised controlled trials (RCTs) are considered the ‘gold standard’ for evaluating the effectiveness of interventions [1]. However, conducting high-quality RCTs may prove to be challenging because of unexpected events that occur ‘en route’, leading to premature discontinuation in an estimated 25% of RCTs conducted in Europe, Canada and Australia [2–4].

Trials may be stopped prematurely for a variety of factors, including unexpected harmful events [5] and greater-than-expected benefits of the intervention manifesting early in the trial [6]. New findings answering the primary research question or raising concerns about the safety of the intervention may also arise [7]. Although these factors play a role in the premature discontinuation of RCTs, the literature suggests that the main reason for terminating a study early is the slow and inadequate recruitment of participants [3, 8, 9]. This, along with evidence indicating that recruitment is adequate and timely in only 50% of RCTs worldwide [10], renders recruitment a key issue. In the UK, several studies have reported similar results in trials funded by two of the UK’s largest funding agencies: the UK Medical Research Council and the National Institute of Health Research (NIHR) Health Technology Assessment (HTA) Programme [9, 11, 12].

An NIHR HTA–funded trial of the anti-depressant sertraline versus cognitive behavioural therapy (CBT) for generalised anxiety (ToSCA) had to be discontinued prematurely because of a failure to recruit. Generalised anxiety disorder (GAD) is common, has distressing symptoms and often is associated with poor general functioning, substance misuse and financial difficulties [13, 14]. The 2011 UK National Institute for Health and Clinical Excellence (NICE) guidelines recommend low-intensity psychological therapies as the best initial treatment, but it is uncertain whether pharmacological or psychological therapy provides the most effective longer-term treatment for those not responding to low-intensity therapies [15]. The NIHR HTA commissioned and funded ToSCA, a UK, multi-site, phase IV RCT aiming to answer this question by assessing the clinical effectiveness at 12 months of treatment with the anti-depressant drug sertraline compared with high-intensity CBT in participants with established GAD, who had failed to respond to a low-intensity psychological intervention. The study outcomes included measures of the effects of anxiety, participant satisfaction and health economic data.

The study set-up started in August 2014, and participant recruitment was anticipated to commence in February 2015. However, participant recruitment was delayed until July 2015 because of several research governance issues, at both a national and a local level, which required consultation with both the lead Clinical Research Network and the Health Research Authority as well as local research and development (R&D) offices for the pilot sites. This was due to a query as to whether participating general practitioner (GP) practices should be registered as individual research sites, which would have been administratively burdensome given that most of the planned 360 trial participants were likely to be registered at different practices. This was addressed with a generic site-specific information form developed by working together with the lead Clinical Research Network and the Health Research Authority, and study-wide R&D approval was received 3 months after initial submission of the full set of documents, although it took a further 2 months to receive local assurances for all of the pilot sites. These delays were very time-consuming and meant that we were not in a position to start work on recruiting participants as planned and initially discussed with the pilot sites, but ultimately they were not the main reason for the failure to recruit as described in the article. The trial aimed to recruit participants via local Improving Access to Psychological Therapies (IAPT) services [16] that served as participating sites. The initial internal pilot phase of the study was planned to run for 12 months and involve four pilot IAPT sites. Unfortunately, however, at 6 months into the internal pilot in January 2016, despite varied attempts to improve participant recruitment, only five patients had been randomly assigned across all of the pilot sites against a target of 40 by this stage. After a monitoring meeting between the study team and the funders, the NIHR HTA decided to discontinue the study and asked the trial team to stop recruiting new participants and to make plans to close down the trial.

Premature discontinuation of RCTs is costly in terms of resources and a failure to answer the underlying research question and is potentially frustrating for the investigators [9, 10, 17] and for the research ethics committees and regulatory agencies that need to devote a considerable amount of time and effort to review protocols [10, 18, 19]. It also raises ethical considerations for patients who have consented to be involved. Therefore, it is very important to evaluate and learn pertinent lessons from previous trials where this has happened.

In light of our experience with the ToSCA trial, we went back to the initial trial design to assess at what points our assumptions about potential recruitment strategies were unsuccessful, and what might have been improved upon or changed in terms of the trial design, in order to give a better chance of a successful outcome. Making this information available to the scientific community may help others to learn from our experience.

## Methods

### Participants’ recruitment

**Fig. 1:**
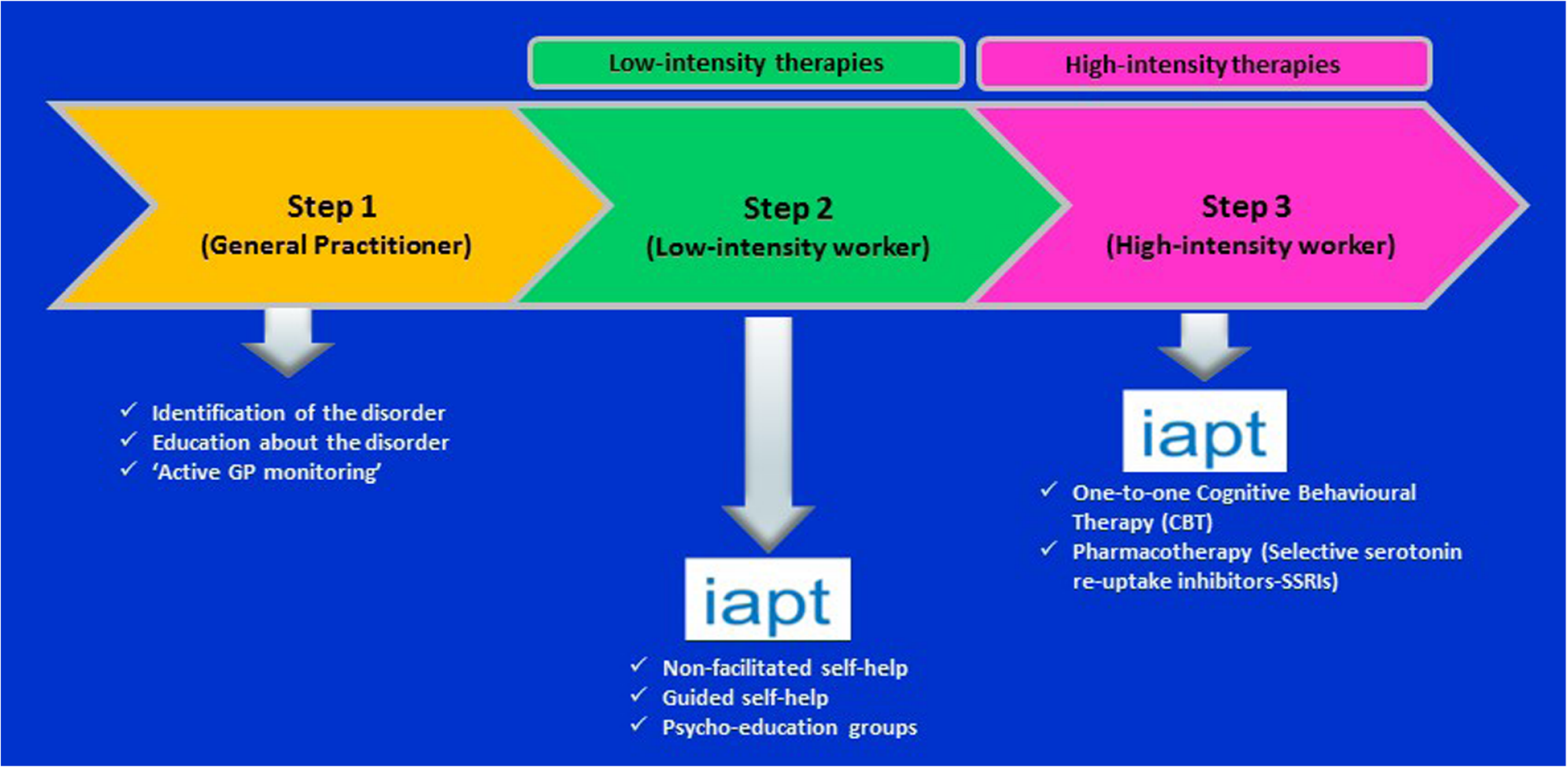
UK National Institute for Health and Clinical Excellence (NICE) stepped care model for generalised anxiety disorder. *Abbreviations*: *GP* general practitioner, *iapt* Improving Access to Psychological Therapies, *SSRI* selective serotonin reuptake inhibitor

Because one of the inclusion criteria to the trial stipulated by the HTA was the failure of patients with diagnosed GAD to have improved with low-intensity psychological interventions delivered by IAPT, the decision was made to recruit trial participants from IAPT sites. Thus, the initial identification of potential participants was carried out by psychological well-being practitioners (PWPs) at the four pilot sites: (i) Camden and Islington (this included the local IAPT service in Kingston as managed by the same National Health Service (NHS) Trust), (ii) Greenwich, (iii) Bristol and (iv) Coventry and Warwick. They were responsible for administering the low-intensity psychological interventions (step 2), which the NICE guidelines indicate should be offered to GAD patients who have not responded to simple education about GAD and active monitoring (step 1). The PWPs would then review patients to see whether they had improved clinically with this intervention and, if not, were in a position to discuss referral to a more intensive psychological intervention (step 3). The trial question was framed around step 3 in the NICE-recommended patient pathway (Fig. 1); it was at this point that the PWPs were asked to raise the topic of the trial with any patients still scoring at least 10 on the GAD-7, a seven-item self-completion questionnaire with very good sensitivity (89%) and specificity (82%) for GAD, which is one of the core measures regularly administered by IAPT services [20]. Some patients who are more severely affected and who have marked functional impairment may bypass step 2 and go directly to step 3. Under this trial protocol, they would not have been approached about taking part in the study, although clinically this might have been appropriate in terms of the interventions being offered within the trial.

Interested patients were provided with a full patient information sheet; with their written consent, a full assessment of their eligibility for the trial was conducted by a member of the research team. Eligible adult participants had to have a primary diagnosis of GAD as diagnosed on the Mini International Neuropsychiatric Interview (MINI) and a positive score of at least 10 on the GAD-7 questionnaire and had to fulfil the other inclusion criteria for the trial [21]. Exclusion criteria included having a comorbid psychotic disorder or bipolar disorder receiving treatment with anti-depressants in the past 8 weeks, having had any high-intensity psychological therapy within the past 6 months, and having current major depression (identified on the MINI) or any comorbid anxiety disorder (also confirmed using the MINI) of more perceived severity than their GAD and any contra-indications for treatment with sertraline ([21], page 8]). Any potential participants expressing thoughts of self-harm at the baseline interview would be screened for significant suicidal ideation by the researcher using an established proforma and referred to their GP or for acute psychiatric assessment if indicated. After giving informed consent and fulfilling all of the eligibility criteria, participants were randomly assigned to either sertraline or CBT via a web-based independent randomisation service.

### Anticipated participant recruitment rates

Patients seen by the PWPs at the low-intensity intervention stage do not all have a definitive psychological diagnosis made, so it is difficult to be sure what number would have definite GAD as distinct from other anxiety disorders or major depression which are often comorbid with GAD. However, they would all have recorded outcomes on the GAD-7 questionnaire, on which a score of at least 10 is suggestive of GAD, as described above. We therefore asked the PWPs to broach assessment for inclusion in the trial with any of their patients who had a GAD-7 score of at least 10 at the end of their low-intensity treatment; the rationale was that a definitive diagnosis would be made when people were being formally assessed for inclusion. Considering retrospective figures from the local IAPT service in Camden and Islington, which had a combined population of 426,000 in 2011–12, we aimed to recruit two participants per month from each of the four pilot sites during the internal pilot with a slower recruitment phase for the first 3 months whilst we were refining our procedures [21]. The overall target for the internal pilot was 90 participants at 12 months and the aim was for 40 at 6 months. It was clear from an early stage in the trial that recruitment was not going as scheduled and a range of interventions were implemented to try to improve this.

### Interventions aiming to improve participant recruitment

Given that successful recruitment to the trial was dependent on recruitment by the IAPT PWPs, we employed a number of interventions aiming to improve recruitment by the PWPs as soon as we became aware of the difficulties with participant recruitment. These included the following:

- Materials produced to help the PWPs keep the study and recruitment in mind. These included reminders of the study eligibility criteria and a crib sheet of points to discuss with possible participants, including a proposed approach to the presentation of the trial and planned interventions as well as suggested responses to queries which might have been raised.
- Funding of lead PWPs to facilitate recruitment. These PWPs were employed by their local IAPT services, where they worked clinically as well as having dedicated, locally funded sessions to facilitate recruitment.
- Regular reminders and meetings with the PWPs about the trial. Members of the research team attended some of these meetings to answer any queries which the PWPs might have about participant recruitment, and several sites used their PWP case management and supervision sessions to highlight patients who might be suitable for the trial at the end of their low-intensity intervention.
- Database searches to identify possible cases. The IAPTUS database, a computerised clinical records system used by all of the IAPT services in this study (IAPTUS; adult version, Mayden) [22], was used to develop an algorithm to identify patients coming to the end of their low-intensity treatment. These patients might have been suitable to approach for the trial if their most recent GAD score was at least 10 and they were known not to be on psychotropic medication. This information was then circulated to the PWPs seeing the identified patients.

### Retrospective analysis of IAPT patient data

Following on from this work during the trial, which did not significantly improve the participant recruitment rates, we performed some retrospective analyses of the IAPTUS database after the study had been terminated, to try to establish whether potential participants who could have been approached about the trial had been missed or whether there were other factors affecting the numbers of people who might be eligible to take part. Data were obtained for all patients assessed during the period from the date that participant recruitment commenced on 1 July 2015 until closure of the ToSCA trial on 8 February 2016 and they were then tracked to completed treatment and discharge. Data were extracted from the Patient Management Software for Psychological Therapies [22] for the IAPT pilot sites in Camden and Islington (including the service located in Kingston) and Bristol. Owing to staff shortages combined with the complexity of the data extraction procedure, data were not extracted for the Greenwich and Coventry & Warwick IAPT sites. Filters were then applied to the data to obtain information relevant to the ToSCA trial. (Further details are available from the study team on request.)

## Results

### Actual recruitment to the ToSCA trial

**Fig. 2:**
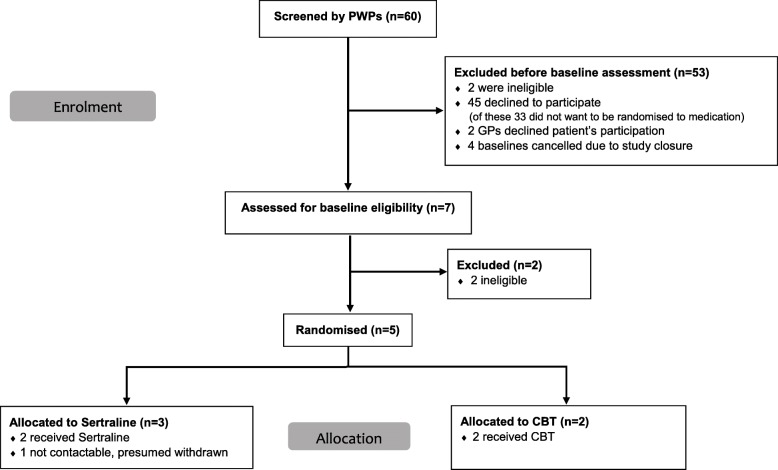
Consort diagram. *Abbreviations*: *CBT* cognitive behavioural therapy, *GP* general practitioner, *PWP* psychological well-being practitioner

During the recruitment period, 60 potential participants were identified by the PWPs as potentially suitable to take part in the trial across the four IAPT pilot sites. In all of these cases, the PWPs discussed the study with the patient and then informed the research team of the results of their discussions. Out of the 60 cases, 53 were excluded from further assessment; the majority (*n* = 45) were excluded because the patients declined to participate (see Consort diagram Fig. 2). The remaining seven underwent baseline assessments, at which two were deemed ineligible for the study (one because GAD was not the psychological problem causing them the most concern and one because they were identified as having comorbid major depression) and five were randomly assigned to either sertraline (*n* = 3) or CBT (*n* = 2). The main reason for patients not wanting to take part in the trial was that they were reluctant to consider being randomly assigned to the medication arm of the trial or they had a definite preference for treatment with CBT or both (33/45).

### Reasons given by PWPs for not identifying potentially eligible patients

It was clear that time and caseload pressures were significant factors for the PWPs, as was the fact that they perceived their clinical work as their priority; given that they were working in a psychological therapy service, they might also have had a subconscious bias towards considering that a psychological treatment for GAD was preferable to medication.

In the PWP meetings attended by the research team to discuss and answer queries about recruitment, the PWPs reported on patients who were potentially eligible for the study but whom they had not identified to the research team. On a few occasions, they had forgotten to discuss the study with the patient; they were sometimes able to rectify this by contacting them and having that discussion. However, the most common issue reported was that the PWP or their supervisor considered that another difficulty described by the patient, such as another anxiety disorder or depression or social difficulties such as severe debt, was the more clinically appropriate problem to treat than any possible or probable GAD. In such cases, the PWP did not consider the patient relevant for the study and so did not discuss it with the patient or identify them to the research team. Although the research team clarified that the research baseline assessment would determine whether the patient met GAD diagnostic criteria and whether their GAD or major depression or a comorbid anxiety problem was the most clinically appropriate and salient problem to the patient to treat, the view expressed by supervisors at the meetings was that it would not be in the best clinical interest of patients to suggest the study to them when the clinical opinion was that treatment targeting GAD would probably not be appropriate.

### Retrospective analysis of IAPT data to identify potential participants

**Fig. 3:**
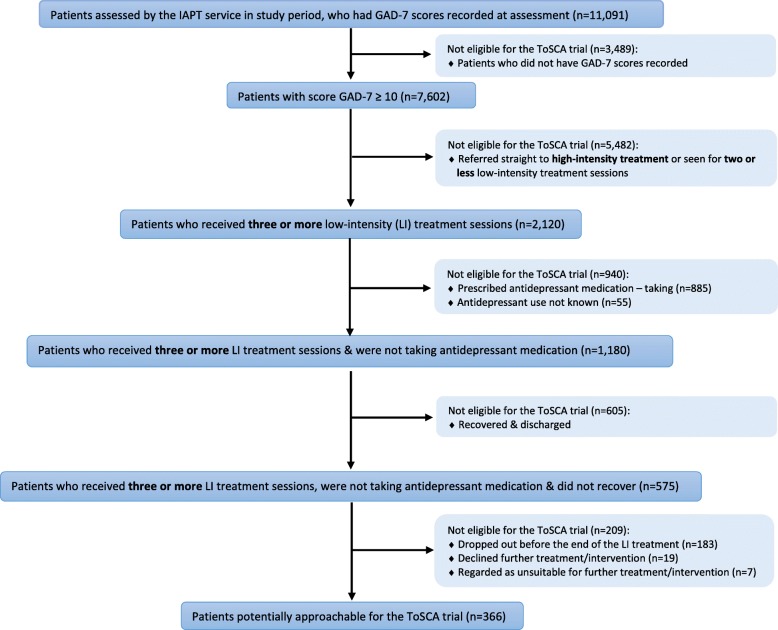
Flow chart showing total database figures for two pilot sites. *Abbreviations*: *GAD-7* Generalised Anxiety Disorder 7, *IAPT* Improving Access to Psychological Therapies

In order to identify the number of potentially eligible and approachable patients for the ToSCA trial, the criteria shown in the flow chart (Fig. 3) were used. During the study period from the date participant recruitment commenced on 1 July 2015 until closure of the ToSCA trial on 8 February 2016, 11,091 patients with an initial GAD-7 result recorded were assessed by the IAPT services across the Camden and Islington (including the IAPT service located in Kingston) and Bristol pilot sites. At this initial clinical assessment, 7602 patients had a score of at least 10 on the GAD-7 questionnaire and 2120 (28%) of these patients had subsequently received three or more low-intensity treatment sessions.

However, 885 out of the 2120 patients who had received three or more low-intensity treatment sessions were recorded in IAPTUS as currently taking anti-depressant medication, and in a further 55 cases, it was not known whether this was the case or not, meaning that 940 patients were not eligible for the study for this reason. Of the remaining 1180 patients who had received at least three low-intensity sessions and were known not to be taking anti-depressants, 605 were recorded as having recovered and thus were not eligible for the trial, leaving 575 potentially eligible patients at this stage.

A further filter was applied to remove patients who had dropped out of low-intensity treatment (*n* = 183), were considered unsuitable for a further intervention (*n* = 7) or had indicated to the PWP that they did not want any further intervention (*n* = 19). This meant that only 366 patients (5% of the original number with an initial GAD-7 score of at least 10) across these two pilot sites would have been potentially approachable for the trial as having a GAD score of at least 10, not being on anti-depressants and having not responded to low-intensity therapy. Although this is considerably more patients than the 44 identified and screened by the PWPs at these two pilot sites during the study period (only 16 of the overall study total of 60 patients were identified and screened at the Greenwich and Coventry and Warwick sites), it is still only around 5% of the 7602 patients with a GAD-7 score of at least 10 at their baseline assessment.

## Discussion

The overall aim of this study was to identify reasons for the poor recruitment to the ToSCA trial which had resulted in its premature closure. Our retrospective database work indicates that only around 12% of potentially eligible patients for the trial were identified by the PWPs at the pilot sites, but the database results also indicate that only a small percentage (5%) of people assessed by the IAPT service with a GAD-7 score of at least 10 and thus possible GAD would have been eligible. In addition, of those patients who were identified by the PWPs in our study as possibly suitable for the trial, three quarters (45/60) declined to take part; the great majority did so because they did not want to be randomly assigned to receive medication.

These findings are in line with the results of a Cochrane review suggesting that poor recruitment to RCTs can be significantly affected by factors influencing the recruiting clinicians [23]. A major problem within the trial appears to have been a low rate of identification of potential participants by the PWPs. We investigated this during the pilot phase and identified several contributing factors, including the fact that the PWPs were clinically stretched and most did not see the research project as a priority for their time. Fletcher et al. [17] suggested that funding-protected research time and having extra staff support [23] could improve recruitment. We managed to secure some additional funding for the lead PWPs (one lead PWP per pilot site) but unfortunately this did not have a significant positive impact on recruitment. Another factor contributing to the low identification rate of potential participants by the PWPs may have been that they are trained to deliver a low-intensity CBT intervention which may have given them a bias, whether conscious or not, towards psychological therapy. There is a potential tension between clinical and research roles when asking clinicians to recruit to research studies [17]. As the recruiting clinicians often also have a gatekeeper role, they may approach only patients whom they personally deem suitable to take part and not all potentially eligible patients for a trial. Additionally, there is evidence for an association between clinicians’ orientation towards research and successful recruitment of patients for clinical trials [23]. Training programmes for clinicians recruiting for RCTs consisting specifically of workshops over one or two consecutive days covering trial-specific and generic research issues may also contribute to enhanced recruitment [24].

Although we thought we had discussed the trial in some detail with the IAPT services involved, it might have been helpful to have discussed the implications of study eligibility criteria more thoroughly in advance with key stakeholders, such as senior clinicians/managers, in order to anticipate potential differences in perspective between clinicians and researchers about the appropriateness for referral to the trial. An alternative might have been to involve members of the research team more directly in participant recruitment (e.g., by placing them within the PWP clinics) but this would have had significant resource implications and was not encouraged by the IAPT services involved.

As only 5% of the 7602 patients passing through the IAPT pilot sites were identified in the database search as potentially eligible for recruitment to the trial, this would have made the whole process very tight. Furthermore, a significant number of the 5% might have not fulfilled all of the study eligibility criteria in terms of not having major depression or having another more significant anxiety disorder, further reducing the population of GAD patients who met eligibility criteria for the study. For example, if one extrapolates from our pilot study, it is possible that three quarters of the 366 deemed potentially eligible may have not wanted to take part, reducing the sample to merely 91 people.

The likelihood that many of the 5% would have another disorder may correlate with the concerns held by the IAPT supervisors about what is the most significant disorder affecting each individual patient. Most GAD is known to be comorbid with other axis 1 disorders [15]. The study criteria would have excluded at baseline assessment all those with comorbid depression and patients considering a comorbid anxiety disorder more significant than their GAD, but it would have been of benefit to get these more accurate figures, not only for recruitment to this trial but also in terms of assessing the relevance of the trial eligibility criteria for the overall service provision for GAD. Shortage of eligible patients (i.e., identification of fewer eligible patients than originally anticipated) has previously been reported as a major obstacle to recruitment in relation to the design and conduct of RCTs [25–27].

In addition, the PWPs stated that their patients were often reluctant to consider a design in which they might be randomly assigned to medication rather than a psychological intervention. Patients’ treatment preferences have been widely reported as key barriers to recruitment [28–31], and a meta-analysis of patient views about receiving psychological or pharmacological treatment for a range of psychiatric disorders indicated a significant preference for psychological treatment [32]. This patient view might have been amplified by the fact that they were already receiving treatment within a psychological therapy service.

Prior experience of treatment has been listed by Stiggelbout and de Haes [33] as a factor shaping patients’ treatment preferences. It has also been demonstrated that the way in which the information about the interventions is presented or ‘framed’ may play a role in shaping patients’ treatment preferences [34, 35]. We attempted to address this factor by suggesting appropriate approaches for the presentation of the trial and the planned interventions as well as suitable responses which the PWPs might give to patient concerns about receiving treatment with anti-depressants, but we had little success. The fact that the medication (i.e., the anti-depressant drug sertraline) was available outside of the trial and that patients were able to get a prescription for it from their GP if they wished might have also played a role in patients’ reluctance to take part in the trial. It would have been very helpful to conduct semi-structured qualitative interviews with both patients and PWPs to further explore these issues around poor recruitment, but unfortunately there was no funding available to do this.

## Conclusions/Future research

An alternative research design may be appropriate to investigate the main clinical question which remains to be answered, namely whether high-intensity CBT or medication has a better longer-term impact for people with clinically significant GAD. This might be carried out by recruiting potential participants from primary care rather than subsequent to referral for a low-intensity treatment in an IAPT setting, as primary care is where initial discussions about whether patients would prefer a pharmacological or psychological approach usually take place and they may be more prepared to accept randomisation to either at this stage. This might mean circumventing the low-intensity treatment indicated at step 2 in the NICE guidelines, and our database work indicated that quite a large number of people with a GAD-7 score of at least 10 improved with three or more sessions of low-intensity therapy (Fig. 3), but an alternative design might be for those in the psychological therapy arm to be initially offered low-intensity therapy and to proceed to high-intensity therapy only if they did not improve. In addition, it would probably be clinically appropriate to ask people being stepped straight up to a step 3 psychological intervention whether they would be interested in taking part in the trial, which was not the case with the design used in the ToSCA trial. Each research design has its pros and cons, but a lesson from this trial might be not to propose or commission a randomised trial with too many restrictions which may affect recruitment as this can affect both participant recruitment and the generalisability of any results.

## Abbreviations

CBT: Cognitive behavioural therapy
GAD: Generalised anxiety disorder
GP: General practitioner
HTA: Health Technology Assessment
IAPT: Improving Access to Psychological Therapies
MINI: Mini International Neuropsychiatric Interview
NICE: National Institute for Health and Clinical Excellence
NIHR: National Institute of Health Research
PWP: Psychological well-being practitioner
R&D: Research and development
RCT: Randomised controlled trial
ToSCA: Trial of Sertraline versus Cognitive behavioural therapy for generalised Anxiety
